# Long-Term Potentiation Promotes Proliferation/Survival and Neuronal Differentiation of Neural Stem/Progenitor Cells

**DOI:** 10.1371/journal.pone.0076860

**Published:** 2013-10-17

**Authors:** Taesup Cho, Jae K. Ryu, Changiz Taghibiglou, Yuan Ge, Allen W. Chan, Lidong Liu, Jie Lu, James G. McLarnon, Yu Tian Wang

**Affiliations:** 1 Brain Research Centre and Department of Medicine, University of British Columbia, Vancouver, Canada; 2 Department of Anesthesiology, Pharmacology and Therapeutics, University of British Columbia, Vancouver, Canada; 3 Translational Medicine Research Center, China Medical University Hospital and Graduate Institute of Immunology, China Medical University, Taichung, Taiwan, Republic of China; Temple University School of Medicine, United States of America

## Abstract

Neural stem cell (NSC) replacement therapy is considered a promising cell replacement therapy for various neurodegenerative diseases. However, the low rate of NSC survival and neurogenesis currently limits its clinical potential. Here, we examined if hippocampal long-term potentiation (LTP), one of the most well characterized forms of synaptic plasticity, promotes neurogenesis by facilitating proliferation/survival and neuronal differentiation of NSCs. We found that the induction of hippocampal LTP significantly facilitates proliferation/survival and neuronal differentiation of both endogenous neural progenitor cells (NPCs) and exogenously transplanted NSCs in the hippocampus in rats. These effects were eliminated by preventing LTP induction by pharmacological blockade of the N-methyl-D-aspartate glutamate receptor (NMDAR) via systemic application of the receptor antagonist, 3-[(R)-2-carboxypiperazin-4-yl]-propyl-1-phosphonic acid (CPP). Moreover, using a NPC-neuron co-culture system, we were able to demonstrate that the LTP-promoted NPC neurogenesis is at least in part mediated by a LTP-increased neuronal release of brain-derived neurotrophic factor (BDNF) and its consequent activation of tropomysosin receptor kinase B (TrkB) receptors on NSCs. Our results indicate that LTP promotes the neurogenesis of both endogenous and exogenously transplanted NSCs in the brain. The study suggests that pre-conditioning of the host brain receiving area with a LTP-inducing deep brain stimulation protocol prior to NSC transplantation may increase the likelihood of success of using NSC transplantation as an effective cell therapy for various neurodegenerative diseases.

## Introduction

A common pathology of a large number of neurodegenerative diseases is neuronal death, and transplantation of neural stem cells (NSCs) to replace the lost neurons is considered a promising potential treatment [1], [2]. However, the sustained survival and neuronal differentiation of exogenously transplanted NSCs, as well as their functional integration into host neuronal circuitry, remain a major challenge [2]. Thus, development of clinically relevant and feasible protocols that can promote proliferation/survival, neuronal differentiation, and functional integration of transplanted NSCs into neuronal networks of the brain is urgently required if exogenously transplanted NSCs are to be utilized as a clinically effective therapy to repair neuronal networks following neuronal damage.

Evidence accumulated in the last few years suggests that activation of N-methyl-D-aspartate receptor (NMDAR), a glutamate receptor subtype, may be involved in regulating proliferation, neuronal differentiation, and survival of newly generated neurons in the hippocampal dentate gyrus (DG) [3], [4]. However, how NMDARs exert these actions remains poorly understood. NMDARs are required to produce certain forms of activity-dependent synaptic plasticity [5]; and NMDAR-dependent long-term potentiation (LTP) and long-term depression (LTD) at glutamatergic synapses in the hippocampus are among the most-well characterized forms of synaptic plasticity [5]. These forms of synaptic plasticity have long been proposed to play critical roles in learning and memory and developmental maturation of neuronal circuits [6], [7]. Furthermore, a recent study has suggested a role for NMDAR-dependent LTP in enhancing proliferation and survival of endogenous neuronal progenitor cells (NPCs) in the hippocampal DG [8]. In addition, evidence accumulated in recent years has also implicated a potential role of NMDARs and possibly synaptic plasticity in regulating neuronal survival and death [9]–[13].

However, whether activation of NMDARs and consequent production of LTP can also promote the survival and neurogenesis of exogenous NSCs transplanted into the brain remain unknown. In the present study we therefore set out to investigate the role of NMDAR-dependent hippocampal LTP in mediating proliferation/survival and neuronal differentiation of endogenous NPCs in the hippocampal DG and, most importantly, of exogenous NSCs transplanted into the hippocampus. The primary goal of the study is to test the potential utility of a LTP-inducing electrical stimulation protocol to promote survival and neurogenesis of NSCs transplanted into the brain, thereby facilitating the clinical use of NSC transplantation for the treatment of a number of neurodegenerative diseases.

## Materials and Methods

### Primary cell culture and neural stem cell isolation

NSCs were isolated directly from the telencephalon, a known developmental precursor of the cerebrum, at E14 from Sprague Dawley (SD) rats. The dissociated telencephalon cells were cultured in Neurobasal media containing B-27 supplement without retinyl acetate (Invitrogen) or N2 supplement (Invitrogen). All cultures contained 20 ng/ml basic fibroblast growth factor (bFGF, PeproTech), 10 ng/ml epidermal growth factor (EGF, PeproTech) and 10 ng/ml leukemia inhibitory growth factor (LIF; Chemicon). The media was changed every 3 days. This procedure resulted in the formation of neurospheres, an aggregate form of NSCs [14], [15]. In order to generate secondary neurospheres, primary neurospheres were dissociated and re-plated onto 24-well dishes for an additional 10–14 days [16]. This second isolation and plating were necessary to obtain pure NSCs, as conventional cell sorting was not feasible due to the lack of a specific NSC surface marker.

Primary cultured hippocampal neurons were prepared and cultured as previously described [10], [17]. Briefly, hippocampal neurons were prepared from E18 SD rats and grown in Neurobasal media (Invitrogen) with B-27 supplement (Invitrogen) containing retinyl acetate, 0.5 mM Glutamax™-1. For initial plating, 25 µM L-glutamic acid (Invitrogen) was added. The media was replaced every 3 days. 10 µM of 5-Fluoro-5′-deoxyuridine (5-FDU; Sigma-Aldrich) was added to inhibit the growth of glial cells in the hippocampal neuron culture.

### Lentivirus production and infection *in vitro*

To produce lentivirus constructs, three different plasmids were transfected into human embryonic kidney (HEK)-293T cells [18]: lentiviral vector plasmid pCMV-containing enhanced GFP, packaging plasmid pR8-2, and envelope plasmid pVSVG. Lentiviral particles were collected from the media once per day for 4 days and centrifuged at 27,500 rpm for 3 hours to concentrate. Lentiviral particles were re-suspended in PBS and tittered by serial dilution on primary cultured cells from the brain. Titers were 6.36×10^8^ TU/ml [18]. For NSC-neuronal co-culture, 3 days after the viral infection, GFP-labeled NSCs were re-plated with dissociated hippocampal neurons and maintained in the hippocampal culture media.

### Retrovirus preparation and intra-hippocampal injection into the DG

Retrovirus was generated using a transient transfection approach in HEK-293T cells. Transfection of the retroviral vectors cytomegalovirus immediate early enhancer-chicken β-actin hybrid-GFP (CAG-GFP), viral proteins (CMV-gag/pol), and capsid (CMV-vsvg) into HEK-293T cells was performed using calcium phosphate (Promega). Virus-containing supernatant was harvested at 48 and 96 hours after transfection, and concentrated by two rounds of ultracentrifugation [19], [20]. All other procedures were the same as the lentivirus concentrate. Titers were 9.51×10^8^ TU/ml. Three days prior to *in vivo* field recording, retrovirus was directly infused onto newly generated cells in the DG through the intra-hippocampal injection manner (3.5 mm posterior to bregma, 2.0 mm lateral to midline, ∼3.6 mm ventral). Rats were anesthetized with sodium pentobarbitol (65 mg/kg, i.p.; MTC Pharmaceuticals) and placed in a stereotaxic frame (David Kopf instruments). Stereotaxic unilateral injection of retrovirus was performed with a stereotaxic-motorized nano-injector (Stoelting) as previously described [21]. The skin was sutured after removing the needle. The animals were allowed to recover before being returned to their home cages.

### *In vivo* field EPSP recordings

Field excitatory postsynaptic potentials (fEPSPs) from the CA1 and DG region of the hippocampus were recorded as previously described [22]–[24]. Five to six-week-old SD rats were anesthetized using sodium pentobarbitol (65 mg/kg, i.p; MTC Pharmaceuticals) and placed into a stereotaxic frame (David Kopf instruments). Supplemental anesthesia was provided hourly at 10% of the initial dose for the duration of the recording. Rectal temperature was maintained at 37±0.5°C during the course of the surgery using a temperature controller (Harvard Instruments). The scalp was opened and separated. Trephine holes were drilled into the skull and bipolar stimulating electrodes (4.0 mm posterior to bregma, 3.0 mm lateral to midline for the CA1 and 7.4 mm posterior to bregma 3.0 mm lateral to midline for the DG) and monopolar recording electrodes (3.5 mm posterior to bregma, 2.0 mm lateral to midline for both the CA1 and DG) were positioned in the CA1 stratum radiatum area or the DG granule cell layer region of the hippocampus, respectively. Final depths of the electrodes were adjusted to optimize the magnitude of the evoked responses. fEPSPs were adjusted to 60% of maximal response size for testing. Stimulation was generated by an analog-to-digital interface (1322A, Axon Instruments) and a Digital Stimulus Isolation unit (Getting Instruments). Pyramidal or granule neuron responses to the Schaffer collateral stimulation or medial perforant path (MPP) were recorded by a differential amplifier (P55 A. C. pre-amplifier, Astro-Med Inc.) and analyzed with WinLTP software (WinLTP Ltd.). Responses were evoked by single pulse stimuli and were delivered at 20-s intervals. A stable baseline was recorded for 30 min. LTP was induced by applying HFS (4 trains of 50 pulses at 100 Hz, 15-s inter-train interval) for the CA1 or the strong theta-patterned stimulation (sTPS, 4 trains of 10 bursts of 5 pulses at 400 Hz with a 200 ms inter-burst interval, 15-s inter-train interval) [25] for the DG. 3-[(±)-2-carboxypiperazin-4-yl]-propyl-1-phosphate (CPP, 10 mg/kg, 90 min before HFS stimulation, Tocris Bioscience) was applied 90 min prior to either sTPS or HFS stimulation to block the induction of LTP.

### Neural stem cell transplantation in the CA1

Two to five hours after CA1 field recording, rats were anesthetized with sodium pentobarbitol (65 mg/kg, i.p; MTC Pharmaceuticals) and replaced into a stereotaxic frame (David Kopf instruments). Stereotaxic unilateral transplantation of GFP-labeled NSCs was performed with a stereotaxic-motorized nano-injector (Stoelting). Injection coordinates for the hippocampus were the same as the recording electrode. GFP-labeled NSCs (5×10^3^, 1 µl, 0.20 µl/min) were slowly injected into the CA1 region of the hippocampus using a 10 µl Hamilton syringe fixed to a 26-gauge needle. After injection, the syringe was left in the place for an additional 5 min and the needle was then slowly withdrawn. The skin was sutured after removing the needle. The animals were allowed to recover and were returned to their home cages.

This study was carried out in strict accordance with the recommendation in the Guide for the Care and Use of Laboratory Animals of the Canadian Council on Animal Care (CCAC). All animal protocols were approved by the Committee on the Ethics of Animal Experiments of the University of British Columbia (Permit Number: A08-0207, A06-0356, A03-0313, B09-0139). All surgery was performed under sodium pentobarbital anesthesia, and all efforts were made to minimize suffering.

### Chemical LTP induction and conditioned medium stimulation

Electrical stimulation of neurons for the induction of LTP is typically used. However, these methods are limited to a localized synaptic area. Therefore, a global application of chemical-induced synaptic plasticity, such chemical LTP (cLTP) was used to reliably induce LTP *in vitro*. NMDAR-dependent cLTP in the culture was induced by brief bath application of high concentration of NMDAR co-agonist glycine (200 µM) to selectively stimulate synaptic NMDARs [10], 200 mM sucrose to stimulate presynaptic release [26]–[28], and 5 µM strychnine to block strychnine-sensitive glycine-gated Cl^-^ receptors [29] in the Mg^2+^-free extracellular solution (ECS, pH 7.35, 140 mM, NaCl, 5.4 mM KCl, 1.3 mM CaCl_2_, 33 mM, 10 mM HEPES 33 mM glucose; 310–320 mOsm). This glycine-induced cLTP protocol has previously been demonstrated to cause a rapid exocytosis of α-amino-3-hydroxy-5-methyl-4-isoxazolepropionic acid receptors (AMPARs) into the synaptic plasma membrane, leading to LTP of AMPAR-mediated excitatory transmission [10], [30]–[35]. Dissociated NSCs from neurospheres were labeled by GFP-containing-lentivirus and then co-cultured with dissociated hippocampal neurons for 8-10 days *in vitro*. The cultured hippocampal neurons alone or co-cultured NSCs with hippocampal neurons were stimulated by a cLTP protocol for 2 min and further incubated for 8 min in the same solution omitting sucrose [10], [35].

Conditioned medium was collected from non-stimulated, PBS-stimulated, or cLTP-induced cultured hippocampal neurons 1 hour after stimulation, and then used for either ELISA analysis of secreted growth factors or stimulation of cultured NSCs alone. Conditioned media was chronically applied every 3 days for 2 weeks.

### ELISA assay

ELISA assays for various growth factors were performed using ELISA kits purchased from Promega (BDNF Emax-, NGF Emax-, and NT-3 Emax immunoassay System). Specifically, 96-well plates (Nunc-Immuno Maxisorp) were pre-coated with anti-monoclonal brain-derived neurotrophic factor (BDNF), anti-polyclonal nerve growth factor (NGF) and neurotrophin-3 (NT-3). They were subsequently incubated with the blocking buffer (goat serum) for 1 hour to prevent non-specific binding. Collected conditioned media from non-, PBS- and cLTP-stimulated hippocampal neurons at various time points were incubated in the pre-coated 96-well plates with agitation at room temperature. The plates were then incubated with anti-polyclonal BDNF, and anti-monoclonal NGF, and NT-3 overnight at 4°C, followed by horseradish peroxidase (HRP)-conjugated secondary antibodies for another 2.5 hours with agitation at room temperature. Each plate was washed 5 times between each step with tris-buffered saline containing 0.05% Tween-20 (TBST, Sigma-Aldrich). The color reaction was stopped by the addition of 1 N hydrochloric acid (HCl) and the absorbance wavelengths of the samples were read on a quant spectrophotometer (Bio-Tec Instrument Company) at 450 nm.

### Antibodies

The following anti-mouse primary antibodies were used against 5-bromo-2′-deoxyuridine (BrdU, 1∶4 for immunofluorescence (IF); Upstate): GFP (IgG, 1∶1000 for IF; Invitrogen), AMPAR subunit, GluR2 (IgG, 1∶1000 for western blotting (WB); Chemicon), nestin (IgG, 1∶200 and 1∶100; Chemicon), neuronal nuclei (NeuN, IgG_1_, 1∶500 for IF; Chemicon), NMDAR subunit, NR1 (IgG2a, 1∶1000 for WB; Chemicon), proliferating cell nuclear antigen (PCNA, IgG2a, 1.500 for IF; Abcam), tropomysosin receptor kinase B (TrkB, IgG_1_, 1∶1000 for WB; BD biosciences), and vimentin (IgG, 1∶1000 and 1∶500; Sigma-Aldrich). Anti-rabbit Primary antibodies were used against β-actin (IgG, 1∶2000 for WB; Abcam), doublecortin (DCX, IgG, 1∶1000 for IF; Abcam), γ-aminobutyric acid_A_ receptor-alpha 1 (GABA_A_R-α1, IgG, 1∶500 for WB; Upstate), glial fibrillary acidic protein (GFAP, IgG, 1∶100 and 1∶80; Sigma-Aldrich), GFP (IgG, 1∶1000 for IF; Invitrogen), GluR1 (IgG, 1∶25 for WB; Calbiochem), β-low density lipoprotein receptor-related protein1 (β-LRP1, lgG, 1∶1000; provided by Dr. Zemin Yao), microtubule associated protein 2 (MAP2, IgG, 1∶1000 for IF; Chemicon) and phosphor-Trk (IgG, 1∶1000 for WB; Cell Signaling). The order of dilution was WB and IF. Anti-mouse and rabbit secondary Alexa Fluor 488 and 555 (IgG, 1∶1000; Molecular probes) antibodies were used for IF, and HRP-linked secondary antibodies were used for WB (1∶10,000; GE Healthcare Biosciences).

### Western blotting

Western blotting was performed as previously described [12], [17]. Cells were washed 3 times with PBS and subsequently harvested with lysis buffer containing the following protease inhibitors: 300 µM 4-(2-aminoethyl)-benzenesulfonyl-fluoride hydrochloride (AEBSF) (Sigma-Aldrich), 10 µg/ml leupeptin (Bioshop), and 10 µg/ml aprotinin (Bayer). The sample pellet was dissolved in the loading buffer and boiled for 5 min. Samples were subjected to SDS-PAGE, and the proteins were transferred onto a PVDF membrane (Millipore), blocked with either 5% skim milk or bovine serum albumin, and probed with relevant antibodies. HRP-conjugated secondary antibodies (GE Healthcare) were used to develop immunoblots. Blots were developed by using ECL detection (GE Healthcare). Band intensities were quantified using ImageJ software (NIH) and normalized to the quantity of either β-actin, a surface marker of β-LRP1, or TrkB as a loading control. All primary antibodies were diluted in TBST and washes were done with shaking between each step.

### Biotinylation of cell surface proteins

Membrane trafficking of AMPAR subunits GluA1 and GluA2 were quantified by surface biotinylation in control or cLTP-stimulated hippocampal neurons as previously described [36]. Cultured cells were tagged with 1 mg/ml sulfosuccinimidy1-6-[biotinamido] hexanoate (sulfo-NHS-LC-Biotin; MJS BioLynx Inc.) for 30 min. Neurons were rinsed 3 times in cold PBS and then harvested in lysis buffer with 1 mM EDTA, 0.5% Triton X-100 and 1% SDS in PBS with three kinds of protease inhibitors (300 µM AEBSF, 10 µg/ml aprotinin, and 10 µg/ml leupeptin). The lysates were subjected to overnight avidin precipitation (280 µg of total protein/60 µl of avidin suspension; Sigma-Aldrich), washed 4 times and subjected to SDS-PAGE. Western blotting was performed as described above.

### Immunocytochemistry and BrdU labeling

Immunocytochemistry was performed on 12 mm round cover glasses (Deckglaser). Cultured cells were washed briefly with PBS. They were subsequently fixed in 4% paraformaldehyde for 10 min and permeabilized with 0.1% Triton X-100 (Sigma-Aldrich) in PBS for 5 min at room temperature. Cells were then blocked in 5% goat serum in PBS overnight at 4°C or 1 hour at room temperature. Goat-raised secondary antibodies were incubated overnight at 4°C or 1 hour at room temperature. For nuclear staining, cells were incubated with DAPI (1∶1000; Sigma-Aldrich) in PBS for 30 seconds prior to mounting on slides in polyvinyl alcohol mounting media with DABCO antifade (Sigma-Aldrich). BrdU was used to detect the NSC proliferation. One day prior to the cLTP induction, 10 µM of BrdU (Sigma-Aldrich) was added to NSCs with or without hippocampal neurons respectively. After cLTP induction, the NSCs were then fixed in 95% ethanol containing 5% glacial acetic acid and followed by incubation in mouse anti-BrdU (1∶4; Upstate) for 1 hour at room temperature. Immunocytochemistry was subsequently performed as described above. Extensive PBS washings were performed between each step.

### Immunohistochemistry

Rats were anesthetized with 65 mg/kg of sodium pentobarbital (65 mg/kg, i.p; MTC Pharmaceuticals) and then transcardially perfused with heparinized cold saline and subsequently perfused with 4% paraformaldehyde in PBS (pH 7.4). The brains were removed from the skull, post-fixed in the same fixative overnight and then dehydrated in 30% sucrose-containing PBS. The brains were then rapidly frozen in powdered dry ice. Coronal sections (30 µm) were cut on a cryostat throughout the hippocampus and stored in the glycerol-cryoprotectant solution. Free-floating sections were processed for single immunohistochemistry as described previously [37], [38]. Briefly, sections were permeabilized by 0.2% Triton X-100 (Sigma-Aldrich) in PBS containing 5% BSA for 30 to 60 min. Slices were then incubated overnight at 4°C with primary and secondary antibodies in 1% BSA-containing PBS one after another. Finally, slices were mounted onto Superfrost/Plus microscope slides (Fisher Scientific) with mounting media (Sigma-Aldrich). For negative controls, immunohistochemistry was performed while omitting incubation of the primary antibody.

### Image quantification and statistical analysis

To analyze *in vivo* retroviral injection in the DG and transplanted NSCs in the CA1, GFP-labeled cells throughout the rostral-caudal extent of the transduced region were counted. We performed statistical analysis using at least 5 sections per rat and then entire CA1 or DG images were acquired using a Leica DMIRE2 microscope. Digitized images were then analyzed using OpenLab 3.7. For measurement of proliferation/survival of NPCs in the DG and transplanted NSCs in the CA1, the numbers of GFP-positive cells were counted. To quantify neuronal differentiation of the NPCs in the DG and of transplanted NSCs in the CA1, the total number of NeuN and GFP double-labeled cells were counted as the ratio of the percentage change from a 0.9% saline-injected control group.

To analyze *in vitro* culture quantitative analysis, five non-overlapping fields in each coverslip (total 3 coverslips for each condition) were randomly selected and images were acquired using either a Zeiss Axioplan-2 microscope equipped with a DVC camera (Diagnostic Instruments, Sterling Heights, MI) or a Leica DMIRE2 microscope. Digitized images were then analyzed using Northern Eclipse software (Empix Imaging) and OpenLab 3.7. Representative images were adjusted to maximize the signal-to-noise ratio. Relative intensities from each fluorophore channel were adjusted to a 50∶50 contribution of signal intensities prior to merging 2 or 3 channel images. For measurement of proliferating NSCs, the numbers of BrdU-positive cells on GFP-labeled NSCs were counted and the percentage of BrdU-labeled cells relative to the total number of GFP-labeled NSCs per field of vision was determined. To quantify neuronal differentiation of NSCs under different treatment procedures, the total number of MAP2 and GFP double-labeled cells were counted as the ratio of the percentage change from a PBS-treated control group. DAPI (1∶1000, Sigma-Aldrich) was applied for the total number of the cells. All quantifications were done in a blinded manner and statistical analyses of One Way ANOVA and *post hoc* testing were carried out as indicated in each figure.

## Results

### The induction of LTP increases proliferation/survival of endogenous NPCs in the subgranular zone of the DG region

**Figure 1:**
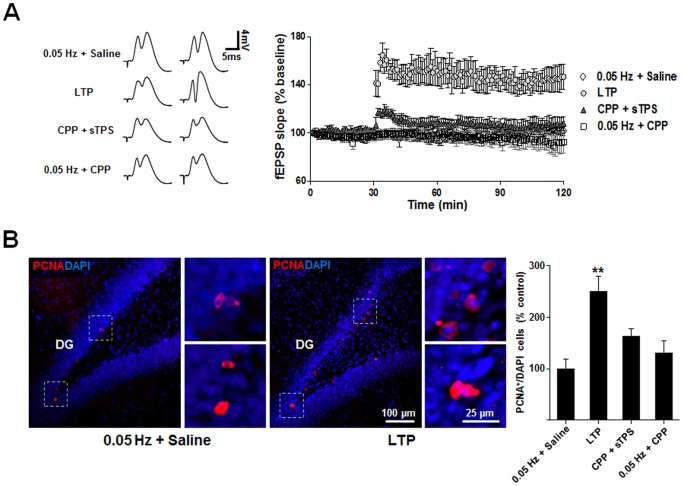
NMDAR-dependent LTP enhances proliferation of NPCs in the DG. (*A*) The induction of LTP in the hippocampal DG requires activation of NMDARs. A stable basal level of fEPSPs was obtained by electrical stimulation of MPP (0.05 Hz) from the DG of anesthetized rats and LTP was induced by application of sTPS through the same stimulation electrode. Representative traces of fEPSP on the left were averages of all individual recordings before and after the establishment of LTP. Systemic application of the competitive NMDAR antagonist CPP (10 mg/kg) prevented the induction of LTP (CPP+sTPS), without affecting basal level of fEPSP (0.05 Hz+CPP). (*B*) LTP increases the total numbers of PCNA-positive immature neurons in the DG. Representative images from coronal sections from non-LTP (0.05 Hz+ saline) and LTP-induced (LTP) rats co-stained with PCNA (red) and DAPI (blue) in the DG area. Examples of doubled-stained cells in boxed areas were shown in higher magnification on the right panels. Bar graph on the right summarizes data from various groups, suggesting enhancement of proliferation by LTP induction. Note CPP pretreatment alone (0.05 Hz+CPP) has no effect on intrinsic proliferation, but prevented LTP-promoted NPC proliferation (CPP+sTPS). **p<0.01, n = 8 or 9. Statistical analysis was performed with One Way ANOVA. *Post hoc* testing revealed a significant difference between the LTP group and other three groups. Data are mean ± SEM. significantly different from each other.

NPCs in the adult DG are one of the major sources of neuronal precursors in the brain [14], [39]-[41]. However, only a small fraction of these cells survive and differentiate into mature neurons. To determine if the induction of LTP promotes proliferation and/or survival of NPCs in the subgranular zone (SGZ) of the DG, we induced NMDAR-mediated LTP in the rat hippocampus. fEPSPs evoked by electrical stimulation of MPP at 0.05 Hz were recorded from the DG region, and after a stable baseline recording was obtained, LTP was induced by sTPS (4 trains of 10 bursts of 5 pulses at 400 Hz with a 200 ms inter-burst interval, 15-s inter-train interval). As shown in Fig. 1 A, this sTPS stimulation reliably elicited LTP of the slope of fEPSPs in the DG region. LTP was mediated by NMDARs as it was completely abolished by a pretreatment with the competitive NMDAR antagonist CPP (10 mg/kg, i.p., 90 min prior to the induction). Control electrical stimulation (0.05 Hz) administered with either 0.9% saline (i.p., 90 min prior to the induction) or CPP did not introduce any change in the baseline fEPSPs (Fig. 1 A). One week after electrical field recordings, the animals were sacrificed and the extent of proliferation of NPCs in the SGZ of the DG was assessed using immunohistochemistry with an antibody against PCNA, a cell cycle marker for proliferation [42]. A significant increase in the total numbers of PCNA-positive NPCs was observed in rats in which LTP was successfully induced (Fig. 1 B, right image), when compared to saline injected rats (0.05 Hz+ saline; Fig. 1 B, left image). This LTP-induced increase in PCNA-positive NPCs was eliminated by the blockade of NMDARs with CPP. Neither 0.9% saline nor CPP injection alone altered the number of PCNA-positive NPCs (Fig. 1 B). These results strongly suggest that NMDAR-dependent LTP can increase the total number of endogenous NPCs in the SGZ of the DG.

### LTP promotes neuronal differentiation of endogenous NPCs in the DG region

**Figure 2:**
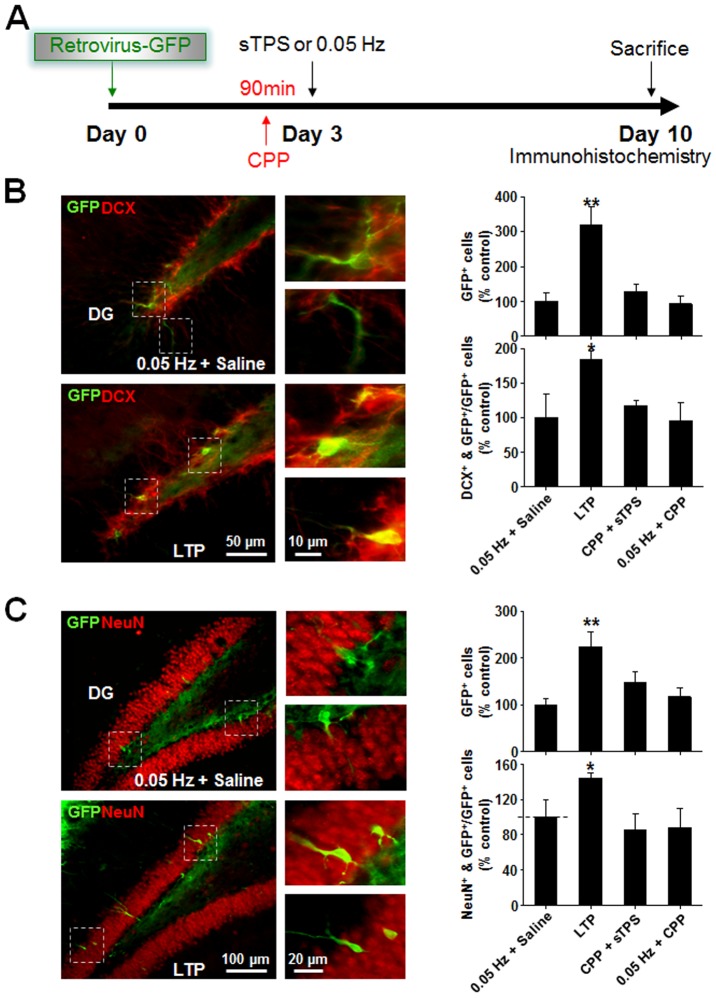
NMDAR-dependent LTP enhances neuronal differentiation and maturation of NPCs in the DG. (*A*) Schematic representation of the experimental design. (*B*) The induction of LTP increases the total number of GFP-labeled NPCs, and DCX and GFP double-labeled immature neurons differentiated from NPCs in the DG. Left: Representative images from coronal sections double-stained with antibodies against an immature neuronal marker DCX (red) and GFP (green) in the DG. Examples of double-stained immature neurons in boxed areas are shown in higher magnification in the panels on the right. Right: Bar graphs summarizing effects of LTP induction on the total number of GFP-positive cells (top panel), and DCX and GFP double-labeled cells (bottom panel) in the DG. *p<0.05 and **p<0.01; n = 8 or 9 in each group. (*C*) The induction of LTP increases the total number of GFP-labeled NPCs, and NeuN and GFP double-labeled differentiated mature neurons in the DG. Left: Representative images from coronal sections double-stained with antibodies against a mature neuronal marker NeuN (red) and GFP (green) in the DG. Examples of double-stained mature neurons in boxed areas are shown in higher magnification in the panels on the right. Right: Bar graphs summarizing effects of LTP induction on the total number of GFP-positive cells (top panel), and NeuN and GFP double-labeled cells (bottom panel) in the DG. *p<0.05 and **p<0.01; n = 8 or 9 in each group. Statistical analyses were performed with One Way ANOVA. *Post hoc* testing revealed a significant difference between the LTP group and other three groups. Data are mean ± SEM.

The increased number of PCNA-positive NPCs following LTP induction may result from enhanced proliferation and/or survival of newly generated NPCs. In addition, LTP may also promote neuronal differentiation of these NPCs. Previous studies have established that NPCs can be specifically labeled by a retrovirus GFP vector [19], [20], [43], [44]. To determine if LTP promotes the survival and neuronal differentiation of NPCs, we labeled hippocampal DG NPCs with a GFP-containing retrovirus (CAG-GFP) via intra-hippocampal injection into the DG area and investigated the effects of LTP induction on GFP-labeled NPC neuronal differentiation and number using an experimental protocol illustrated in Fig. 2 A. LTP was induced by sTPS 3 days following virus infection. One week following LTP induction, the survival of NPCs was assessed by counting the total number of GFP-labeled cells, while neuronal differentiation was estimated by examining the expression of either DCX, an immature neuronal marker [45]–[48], or NeuN, a mature neuronal marker [49] in GFP-expressing NPCs. As shown in Fig. 2 B and *C*, LTP induction significantly increased the total number of GFP-labeled cells in a NMDAR-dependent manner, suggesting increased NPC survival. The induction of LTP also resulted in a significant increase in the number of DCX-positive GFP-NPCs, a result not observed when using basal, non-LTP-inducing stimulation (0.05 Hz+ saline; Fig. 2 B), and one that could be prevented by pretreatment of the rats with the NMDAR competitive antagonist CPP (Fig. 2 B). Thus, LTP appears to promote neuronal differentiation of NPCs into immature neurons. In addition, LTP induction was also associated with a significant increase in the number of NeuN-positive GFP-NPCs (Fig. 2 C). In accordance with a general pattern of NPC migration into the granule cell layer from the SGZ as they differentiate and mature [50], these fully differentiated mature GFP-NeuN-positive neurons were primarily located in the inner granule cell layer (Fig. 2 C). Furthermore, LTP-induced neuronal maturation and migration was eliminated by CPP application (Fig. 2 C). Since neither 0.9% saline nor CPP injection in the absence of LTP induction altered the number of GFP-, DCX-, and NeuN-positive cells, the results indicate that LTP promotes neuronal maturation of GFP-NPCs.

### LTP enhances neurogenesis of exogenous NSCs transplanted into the hippocampal CA1 region

**Figure 3:**
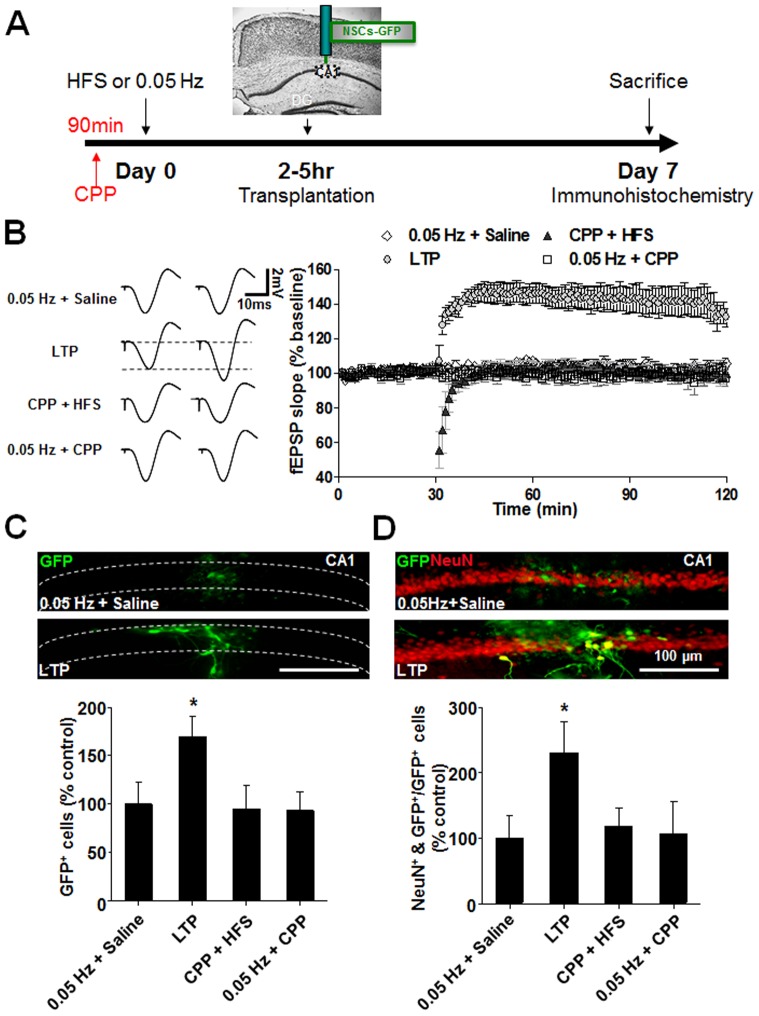
The induction of LTP increases neuronal differentiation of exogenous NSCs transplanted into the CA1 region. (*A)* Schematic representation of the experimental design. (*B*) NMDAR-dependent LTP in the hippocampal CA1 region was reliably induced by HFS of the Schaffer collateral inputs in anesthetized rats. Left: Representative traces of fEPSP were averages of individual recordings taken before and after the establishment of LTP. Right: Systemic application of the competitive NMDAR antagonist CPP prevented the induction of LTP (CPP+HFS), without affecting basal level of fEPSP (0.05 Hz+CPP). (*C*) Representative images from coronal sections show LTP (LTP) enhanced the total GFP (green)-positive cells in the CA1 compared with the control group (0.05 Hz+ saline). Bar graph below summarizes data from each group of rats. LTP increased the total numbers of transplanted NSCs. (*D*) Representative images from coronal sections double-stained for GFP and NeuN showing LTP (LTP) increased numbers of NeuN-positive (red) NSCs (green) in the CA1 region, compared with the control group (0.05 Hz+ saline). Bar graph below summarizing data of LTP enhancement of neuronal differentiation. *p<0.05, n = 7, 7, 10, and 8. Statistical analyses were performed with One Way ANOVA. *Post hoc* testing revealed a significant difference between the pre-conditioning LTP group and other three groups. Data are mean ± SEM.

The increased proliferation/survival and neuronal differentiation of endogenous NPCs prompted us to next determine if LTP has similar actions on exogenously transplanted NSCs. To this end, we chose to examine the effects of LTP in NSCs transplanted into the hippocampal CA1 (but not the DG) of anesthetized rats (Fig. 3 A), an approach chosen in order to minimize any potential influences on the transplanted NSCs from endogenous stem cell niches present in the DG [51]. Like DG LTP, CA1 LTP is a well-characterized NMDAR-dependent form of synaptic plasticity [5], and one which can be reliably induced *in vivo* [22]. As shown in Fig. 3 B, LTP of CA1 fEPSPs was reliably induced by a short train of HFS (4 trains of 50 pulses at 100 Hz, 15-s inter-train interval) of the Schaffer collaterals. LTP was an NMDAR-mediated phenomenon as it was abolished by the NMDAR antagonist CPP (10 mg/kg, i.p., 90 min prior to the induction). Control stimulation (0.05 Hz) following either 0.9% saline (i.p., 90 min prior to the induction) or CPP injection did not introduce any significant alteration in the basal level of fEPSPs (Fig. 3 B). NSCs derived from embryonic cultures were pre-labeled using a GFP-containing lentivirus prior to being transplanted into the CA1 region 2–5 hour following LTP induction. Effects of LTP on proliferation/survival and neuronal differentiation of transplanted NSCs were then examined by quantifying both the total number of GFP-positive transplanted cells and percentage of neuronal marker NeuN-positive GFP cells, respectively, within the CA1 region 7 days after transplantation. As shown in Fig. 3 C, the number of GFP-positive transplanted cells was significantly higher in the CA1 region stimulated with our LTP protocol, when compared to the CA1 region stimulated with a non-LTP-inducing control protocol (0.05 Hz+ saline). Moreover, injection of CPP alone (10 mg/kg, i.p.) had no effect on the number of GFP-positive cells (0.05 Hz+CPP), but did prevent the LTP-induced increase (CPP+HFS). Thus, the induction of LTP increased proliferation/survival of the transplanted NSCs. As shown in Fig. 3 D, immunohistochemical staining also revealed that the induction of CA1 LTP caused a significant increase in the proportion of NeuN-positive transplanted GFP cells in the CA1 region, suggesting increased neuronal differentiation of exogenously transplanted NSCs. Application of CPP prior to LTP induction abolished LTP-increased neuronal differentiation (CPP+HFS). In contrast, CPP application alone (0.05 Hz+CPP) had little effect on the basal neuronal differentiation as it did not alter the proportion of NeuN-positive NSCs in comparison with a saline application group (0.05 Hz+ saline). Thus, the induction of NMDAR-dependent LTP promotes proliferation/survival and neuronal differentiation of exogenously transplanted NSCs in the CA1 region.

### LTP increases neurogenesis of NSCs in cultures *in vitro*

**Figure 4:**
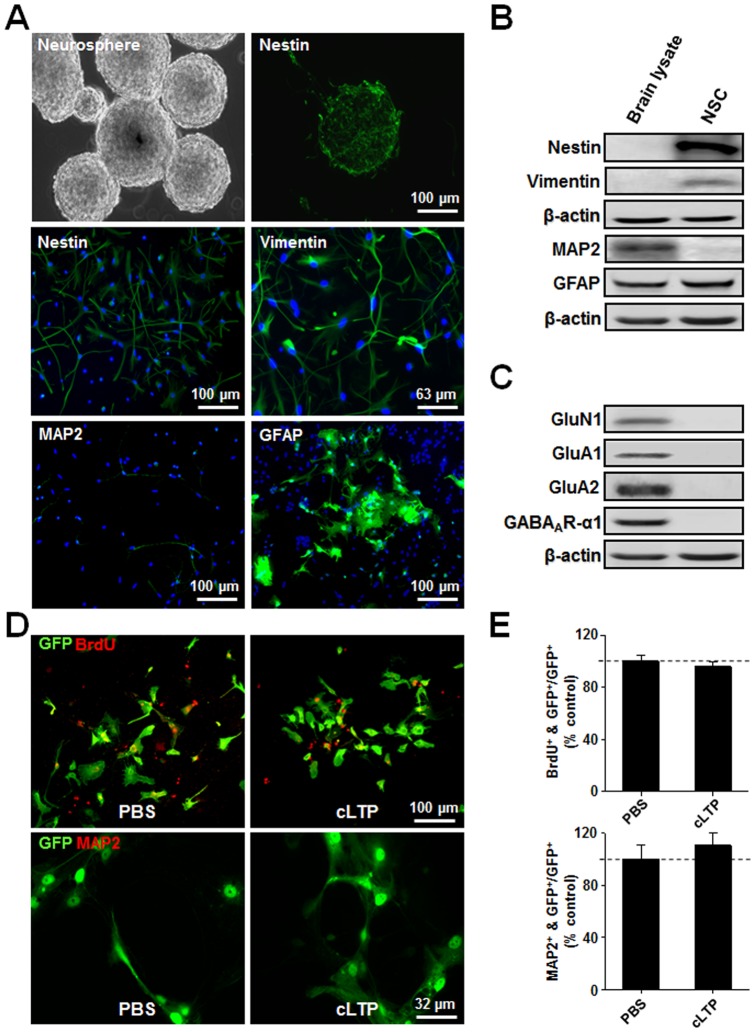
Isolation and characterization of NSCs in cultures. (*A*) Immunostaining characterization of neurosphere and dissociated NSCs. Top two panels show NSCs in neurospheres isolated and characterized from E14 telencephalon. The representative phase contrast image on the left showing the formation of neurospheres and representative immunostaining image on the right showing that majority of cells in the neurosphere express nestin, a NPC marker. The remaining 4 panels are immunostained images of individual NSCs dissociated from neurospheres doubled stained with a nuclear marker DAPI (blue) and one of various cell type markers (green): nestin, vimentin (NPCs), MAP2 (mature neuron) or GFAP (radial glia and/or progenitors). (*B*) and (*C*) Biochemical characterization of NSCs. Western blots of lysates of the adult rat brain, or NSCs grown in media were sequentially probed with various cell type-specific antibodies against nestin, vimentin, MAP2 and GFAP (*B*), and various ionotropic neurotransmitter receptor GluN1, GluA1, GluA2 and GABA_A_R-α1 subunits (C). (*D*) The LTP-inducing treatment does not affect either proliferation or neuronal differentiation of NSCs in the absence of neurons in the cultures. Representative images of GFP-labeled NSCs (green) immunostained BrdU or MAP2 (red). Bar graphs of the grouping data on the right (*D*) showing that treatment of pure NSCs in cultures with the cLTP-inducing protocol did not alter either BrdU (upper panel) or MAP2 (lower panel). *p<0.05; n = 5 in each group. Statistical analyses were performed with Student's t-test. Data are mean ± SEM.

Next, we sought to investigate the underlying mechanisms by which LTP promotes proliferation/survival and neuronal differentiation of NSCs, using mixed NSC and neuronal cultures (NSC-neuron co-culture) used in recent studies [15], [52]. NSCs were isolated from neurospheres derived from E14 rats, then dissociated and re-plated for an additional 10-14 days (Fig. 4 A). Immunocytochemical staining of both neurospheres and dissociated NSCs confirmed that the isolated cells have characteristics of immature NSCs. As shown in Fig. 4 A, the vast majority of the isolated cells (labeled with nuclei fluorescent dye DAPI) expressed type IV intermediate filament protein nestin, a protein marker for NPCs [53], and type III intermediate filament protein vimentin, a protein marker for mitotically active [54], neural progenitors [55] and radial glia [56]. Further supporting their identity as immature NSCs, we found that the vast majority of these neurosphere-derived cells did not express MAP2, a protein marker for fully differentiated mature neurons (Fig. 4 A and B) [57]. Consistent with recent reports that both NSCs and progenitors can express intermediate filament protein GFAP, a protein marker for radial glia [58]-[60], we also observed that these cells expressed GFAP (Fig. 4 A and B). Thus, our immunocytochemical characterization demonstrated that the neurosphere-derived cells isolated in the present study appear to be GFAP-positive NSCs that are immature and capable of self-renewal and proliferation.

The identity of these cells as NSCs was further confirmed with Western blotting of the cellular lysate using antibodies against various cell-type specific markers. Consistent with the immunocytochemistry results, NSCs express nestin, vimentin and GFAP, but not MAP2 (Fig. 4 B), in contrast to adult brain lysate, which expresses mature neuronal MAP2 and glia marker GFAP proteins, but not the immature cell markers nestin and vimentin (Fig. 4 B). To further confirm that these NSCs did not fully differentiate into functional neurons, we also examined their expression of ligand-gated ionotropic glutamate and GABA_A_ receptors (GABA_A_Rs), which, in the vast majority of synapses in fully differentiated neurons, mediate excitatory and inhibitory synaptic transmissions, respectively [5]. As shown in Fig. 4 C, in contrast to the brain lysates, NSCs did not have detectable levels of NMDARs subunit GluN1, AMPA subtype glutamate receptor subunits GluA1 or GluA2, or GABA_A_R α1 subunits. These results further support the idea that NSCs isolated from neurospheres contains few fully differentiated functional neurons.

Following isolation and characterization of NSCs, we investigated the effect of LTP on proliferation and neuronal differentiation of these NSCs using a well-characterized glycine-based cLTP stimulation protocol (see method sections for details) [10], [35]. In this set of experiments, proliferation of NSCs was evaluated by quantifying the number of cells incorporated BrdU after cLTP induction. BrdU is a thymidine analog that can be specifically incorporated into the DNA of dividing cells during the S-phase, and has been widely used for labeling proliferating cells in studies of neurogenesis [61], [62]. Neuronal differentiation was assayed with immunofluorescent staining of neuronal marker MAP2. As shown in Fig. 4 D and E, the glycine stimulation protocol had no obvious effect on either proliferation or neuronal differentiation in NSC cultures. These results are unsurprising, as LTP can only be induced in mature neurons which possess glutamate-mediated excitatory synaptic transmissions.

**Figure 5:**
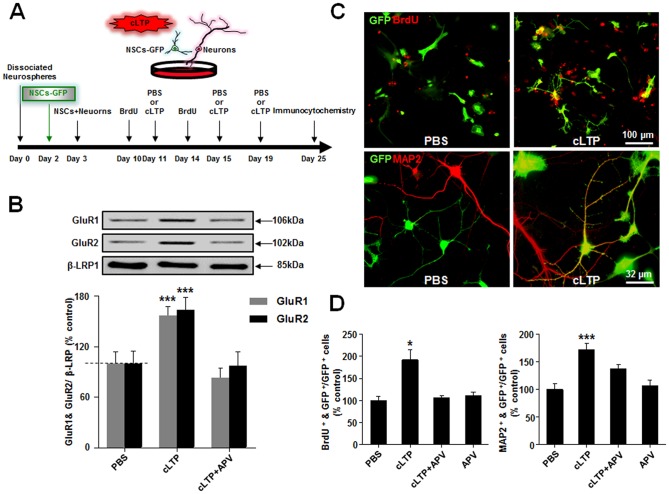
Chemical LTP enhances proliferation/survival and neuronal differentiation in NSC-neuron co-cultures. (*A*) Schematic representation of the experimental design. GFP-labeled NSCs were co-cultured with dissociated hippocampal neurons as described in the methods. (*B*) The cLTP-inducing protocol reliably induces LTP in co-cultures. Western blotting of biotinylated plasma membrane proteins revealed that in comparison with control (PBS), the cLTP-inducing treatment (cLTP) resulted in a specific increase in the level of GluA1 and GluA2 subunits of AMPARs, but not β-LRP1 (as a plasma membrane protein control), expressed on the plasma membranes 10 min after the treatment, and the increase was prevented by 50 µM NMDAR blocker APV (cLTP+APV), confirming the successful induction of NMDAR-dependent LTP. ***p<0.001, n = 7. (*C*) Representative Immunostaining images showing enhanced BrdU (top panel, red) and MAP2-positive (bottom panel, red) GFP-labeled NSCs following cLTP induction in co-cultures. (*D*) Bar graph summarizing data from multiple experiments shown in C. n = 5 and *p<0.05 for left panel; and n = 10 and ***p<0.001 for right panel. Statistical analyses were performed with One Way ANOVA. *Post hoc* testing revealed a significant difference between the cLTP group and other two groups (*B*) or three groups (*D*). Data are mean ± SEM.

Then, we subsequently used NSC-neuron co-cultures to examine LTP effects (Fig. 5 A). In order to facilitate the identification of NSCs from fully differentiated neurons in the co-cultures, we pre-labeled these NSCs using lentivirus vectors containing GFP 3 days before co-culturing them with dissociated embryonic hippocampal neurons. Both dissociated NSCs and neurospheres were infected by lentivirus-GFP at high efficiency (approximately 73%). Unlike in pure NSC cultures, glycine-based cLTP protocol stimulation reliably induced LTP in NSC-neuron co-cultures. Thus, as shown in Fig. 5 B, surface biotinylation assays revealed that glycine stimulation resulted in a rapid and significant increase in the amount of both GluA1 and GluA2 subunits of AMPA receptors (AMPARs) on the membrane surface [10], [35]. This increase in surface AMPARs is not a result of general increases in vesicle membrane fusion, as it was not associated with a detectable change in the amount of membrane protein β-LRP1 (Fig. 5 B). Moreover, a glycine-induced increase in cell-surface expression of AMPARs was prevented by blockade of NMDARs with competitive NMDAR antagonist, (2*R*)-amino-5-phosphonopentanoate (D-APV, 50 µM) (Fig. 5 B). Thus, a glycine-based cLTP stimulation protocol could reliably induce LTP in the NSC-neuron co-cultures. As we expected, cLTP induction was able to significantly increase the number of both BrdU- (Fig. 5 C, upper panel and Fig. 5 D, left panel) and MAP2-positive NSCs (Fig. 5 C, bottom panel and Fig. 5 D, right panel) and importantly, an increase in both BrdU- and MAP2-labeled NSCs could be prevented by D-APV (Fig. 5 D). Neither a PBS treatment (as a control) nor D-APV alone had any detectable effect on proliferation and neuronal differentiation of NSCs (Fig. 5 D). Thus, our results suggest that the induction of cLTP with glycine in neurons promotes both proliferation and neuronal differentiation of NSCs co-cultured with neurons.

### BDNF plays a critical role in mediating LTP-promoted neurogenesis of NSCs in NSC-neuron co-cultures

**Figure 6:**
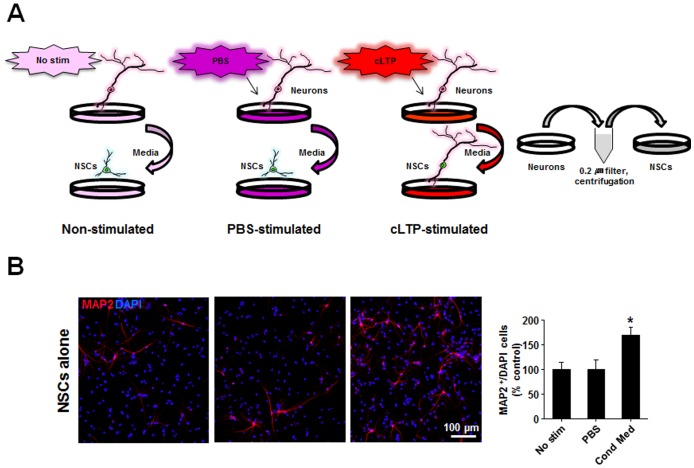
Conditioned media from cLTP treated neuronal cultures increases neurogenesis of NSCs. (*A*) Schematic representation of the experimental design. Conditioned media from non-, PBS- or cLTP-stimulated protocol-treated hippocampal cultures were collected and filtered before their use to treat NSCs in cultures. (*B*) Representative images of NSCs doubled stained with a nuclear marker DAPI (blue) and neuronal marker MAP2 (red) showing increased numbers of MAP2 (red) staining in cultured NSCs alone compared with a control group (non- and PBS-stimulated media). Data from multiple experiments were summarized in the bar graph on the right. *p<0.05, n = 6. A statistical analysis was performed with One Way ANOVA. *Post hoc* tests revealed a significant difference between conditioned-media stimulated group and control group. Data are mean ± SEM.

The requirement for the presence of neurons to enable cLTP-increased proliferation and neuronal differentiation of the cultured NSCs suggests that the effects of cLTP are either from physical contact between NSCs and neurons, or from one or more diffusible factors from the cLTP-stimulated neurons. To differentiate between these two possibilities, we treated the pure NSC cultures with conditioned media from non-, PBS-, or cLTP-stimulated neuronal cultures (Fig. 6 A). As shown in Fig. 6 B, conditioned media from neuronal cultures that experienced LTP induction significantly increased the number of MAP2-positive NSCs, in comparison with conditioned media from either non- or PBS-stimulated controls. These results strongly suggest that the cLTP effects observed in the NSC-neuron co-cultures may not be the result of direct physical contact between the two cell types, but instead, following the induction of cLTP neurons may secrete some diffusible factors that promote proliferation and neuronal differentiation of the co-cultured NSCs.

**Figure 7:**
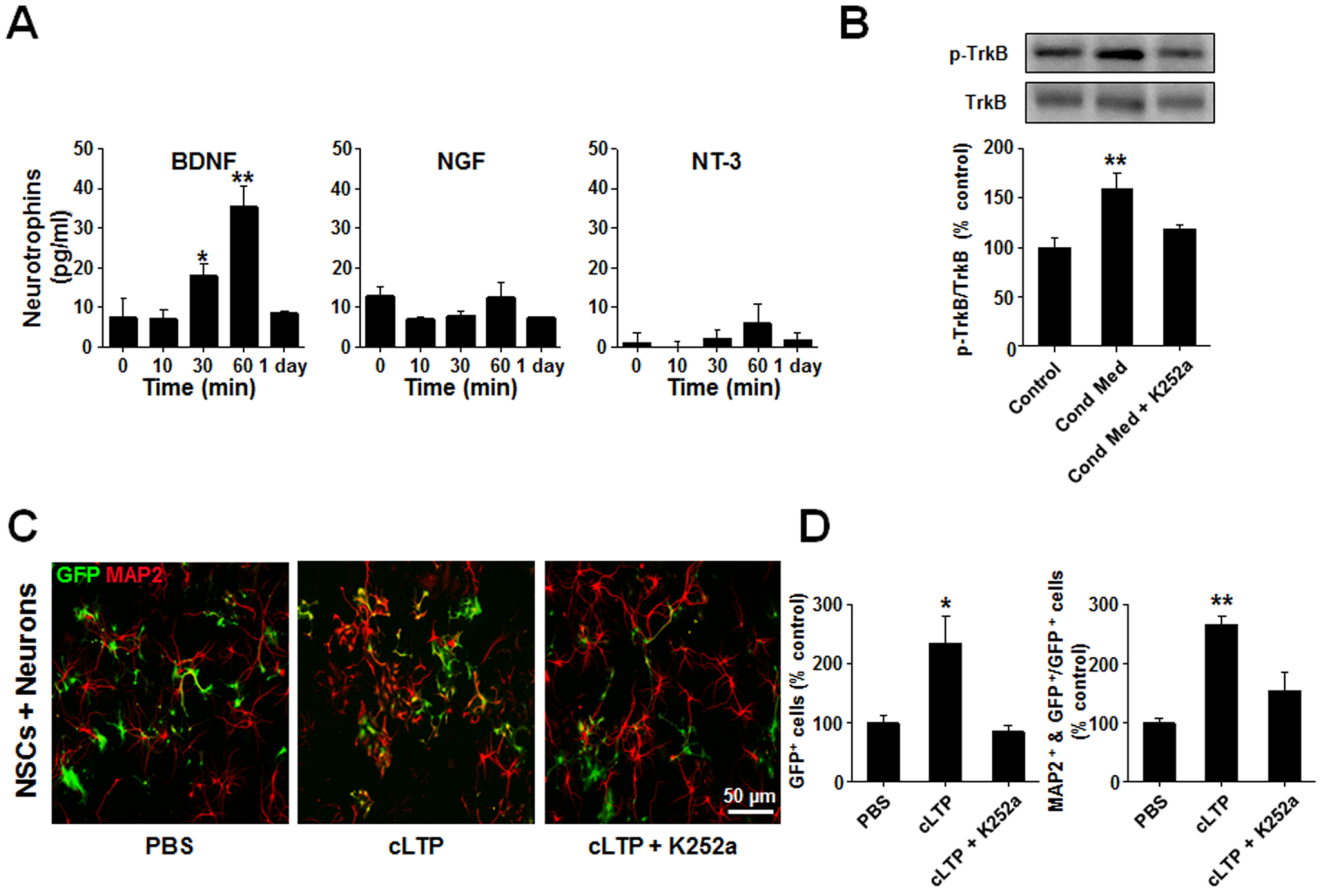
LTP promotes neurogenesis of NSCs in cultures at least in part through BDNF-TrkB system. (*A*) cLTP increases the BDNF production in hippocampal cultures. Conditioned media was collected at 0, 10, 30, 60 min, and 1 day following cLTP induction. ELISA assays reveal a significant increase in the level of BDNF, but not NGF or NT-3, at 30 min and 1 hour following cLTP induction. *p<0.05, **p<0.01, n = 5 for each group. (*B*) cLTP-induced conditioned media activates membrane surface TrkB receptors in cultured NSC alone. Western blotting of surface biotinylated TrkB in NSCs sequentially probed with anti-TrkB tyrosine phosphorylation (p-TrkB) and anti-TrkB (TrkB) antibodies showing the increased level of receptor tyrosine phosphorylation by conditioned media from hippocampal cultures treated with cLTP protocol (cLTP), but not these treated with PBS (Control) and the increased phosphorylation was prevented in the presence of TrkB receptor inhibitor K252a (cLTP+200 nM). Data from 4 individual experiments were summarized in the bar graph at the bottom. **p<0.01, n = 4. (*C*) and (*D*) cLTP enhanced neurogenesis of NSCs in NSC-neuron co-cultures requires activation of TrkB receptors. Representative images (C) of GFP-labeled NSCs immunostained with MAP2 (red) illustrate cLTP increased, in a TrkB dependent fashion, both the total number of NSCs (green; summarized in the left Bar in D) and MAP2-positive NSCs (yellow; summarized in the right bar graph in D). **p<0.01, n = 6. Statistical analyses were performed with One Way ANOVA. Post hoc tests revealed a significant difference between conditioned media and other two group, and (*D*) the LTP group and two other groups. Data are mean ± SEM.

Several growth factors have previously been shown to promote NSC neuronal differentiation. Therefore, we next investigated if one or more of these growth factors could be involved in the cLTP-induced proliferation and neuronal differentiation of NSCs. Using an ELISA assay, we examined the concentration of three growth factors previously implicated in stimulating NSC neuronal differentiation; BDNF, NGF and NT-3 [63]–[68], in the media obtained from the neuronal cultures at 0, 10, 30, 60 min, or 1 day following cLTP induction. We found that BDNF levels, but not NGF or NT-3, were significantly elevated at 30 and 60 min following cLTP induction (Fig. 7 A), suggesting BDNF may play a critical role in mediating the effects of cLTP on NSCs.

In order to investigate this idea directly, we next treated pure NSC cultures with conditioned neuronal culture media obtained 60 min following cLTP induction and examined if it could stimulate TrkB receptors in these NSCs by measuring the level of TrkB receptor tyrosine phosphorylation, an indication of increased TrkB receptor activation [67], [69]–[71]. Western blotting with anti-phopho-TrkB (p-TrkB) antibody revealed an increase in the tyrosine phosphorylation levels of TrkB receptors expressed on the plasma membranes of NSCs and moreover, this conditioned medium-induced increase could be blocked by the application of the tyrosine kinase inhibitor, K252a [72] (Fig. 7 B). Finally, to further support the critical role of BDNF in mediating effects of cLTP on NPCs in NSC-neuron co-cultures, we observed that inhibition of TrkB receptor activation with bath application of K252a prevented the cLTP-induced increase in both the number of GFP-positive, as well as the neuronal differentiation, of NSCs (Fig. 7 C and D). Thus, BDNF released from neurons and its consequent activation of TrkB receptors on NSCs may, at least in part, be responsible for the observed effects of cLTP on NSCs.

## Discussion

In the present study, we showed that the induction of NMDAR-mediated hippocampal LTP promotes proliferation/survival and neuronal differentiation of both endogenous NPCs in the DG and exogenous NSCs transplanted into the CA1 in the rat hippocampus. Furthermore, using *in vitro* NSC-neuron co-cultures, we demonstrated that LTP-induced enhancement of neurogenesis may be, at least in part, mediated through the BDNF-TrkB signaling pathway. These findings strongly suggest that the induction of LTP by either electrical or chemical stimulation may be an efficient way to prolong the proliferation/survival and to promote neuronal differentiation of NPCs/NSCs, and thereby facilitate their anatomical and functional integration into neuronal networks. In particular, given that a brain electrical stimulation device that has been clinically used in deep brain stimulation (DBS) [73]–[75] may be used to induce LTP prior to NSC transplantation, this study may open the possibility of using a LTP-inducing protocol to improve the efficacy of the clinical use of NSC transplantation as a cell replacement therapy to repair or restore damaged neuronal networks in both acute and chronic neurodegenerative diseases.

The adult mammalian central nervous system contains a population of NSCs [14], [39]–[41]. Increasing evidence suggests that certain areas of the adult brain may maintain some ability to generate new neurons and under certain conditions, these newly-generated neurons in the adult brain are able to differentiate into functionally mature neurons and integrate into existing neural circuitry [44], [76]–[78]. It is therefore theoretically possible for these newly generated neurons to functionally compensate for neuronal loss in injured brain regions under pathological conditions [44], [79]. However under normal conditions, the number of these newly-generated cells in the adult brain is extremely low, and their functional maturation and integration into existing neuronal circuits can be further compromised by unfavorable environments in the diseased brain. As a result, intrinsic neurogenesis may be insufficient to compensate for continuous neuronal loss as a natural consequence of ageing throughout life, and/or for pathological neuronal injury in the brain [80]-[84]. In order to overcome these issues, the clinical use of exogenously transplanted NSCs has been considered as an alternative stratagem to repair neuronal networks following neuronal damage. However, neuronal differentiation and sustained survival, two critical conditions required for these exogenously transplanted NSCs to functionally integrate into host neuronal circuitry, remain a big challenge [2].

LTP has previously been suggested to have a potential role in promoting intrinsic neurogenesis by enhancing proliferation and survival of endogenous NPCs. For instance, previous studies reported that the induction of LTP increases numbers of new neurons in the DG [8], [85], [86], Although the underlying mechanisms remains unclear, accumulating evidence suggests that this may be primarily caused by the LTP-enhanced NPC proliferation and survival, but not neuronal differentiation [8], [85]. Moreover, a recent study has further shown that LTP enhanced survival of newly-generated neurons may only occur within a narrow critical period [87]. However, whether LTP can improve proliferation/survival and neuronal differentiation of exogenously transplanted NSCs has not previously been addressed. Our results strongly imply that the induction of LTP may produce a dramatic enhancement of proliferation/survival and neuronal differentiation of not only endogenous NPCs, but also exogenously transplanted NSCs. Consequently, LTP may have profound implications in improving the efficacy of NSC-based cell replacement therapies.

While the details of how the induction of LTP promotes neurogenesis remain not fully understood, our results point towards an NMDAR-dependent release of neurotrophic factors including BDNF from host neurons surrounding NPCs/NSCs as playing a significant role. These findings are consistent with several previous studies that suggested a role for NMDARs and BDNF in promoting neurogenesis [4], [88], [89]. Although some previous studies have shown that the blockade of NMDARs increases the birth of neurons in the DG GCL [90], [91], accumulating evidence strongly support a critical role of NMDARs in promoting both proliferation/survival and neuronal differentiation of NPCs [3], [4]. In support of a similar role of NMDARs in LTP-mediated neurogenesis, we observed that a LTP-induced increase of proliferation/survival and neuronal differentiation of both endogenous NPCs and exogenously implanted NSCs was prevented by pretreatment with a NMDAR blocker. In addition, using a co-culture system, we further demonstrated that cLTP produced by selective activation of synaptic NMDARs was sufficient to mediate most, if not all, effects of LTP on NPCs/NSCs, suggesting that NMDAR activation is not only required, but also sufficient to promote the neurogenesis of NPCs/NSCs. Although Joo and colleagues (2007) suggested a critical role for NMDARs expressed on the NPCs in increasing neurogenesis of these cells [4]; we did not observe any detectible level of NMDARs expression in our pure NSC cultures. In support of the lack of functional NMDARs in this system, we observed that a cLTP stimulation protocol was capable of promoting the neurogenesis in the NSC-neuron co-cultures, but failed to do so in the pure NSC cultures. These results strongly suggest that the LTP-induced neurogenesis observed in our study is primarily mediated by activation of NMDARs in host neurons, but not in NSCs themselves.

The ability of conditioned media from cLTP-induced hippocampal neurons to promote neurogenesis of pure NSC cultures indicates that the neurogenesis promoting effects do not require a physical contact between NSCs and neurons, but rather a critical involvement of cLTP-induced release of neurotrophic factors from neurons, that in turn diffuse to and exert effects on the nearby NSCs. Consistent with this conjecture, we revealed that cLTP can increase the extracellular level of BDNF in neuronal cultures and that TrkB activation is required for LTP-induced neurogenesis in NSC-neuron co-cultures, providing strong support that BDNF is one of the critical neurotrophic factors. This finding is in agreement with several previous studies that reported the role of BDNF in regulating proliferation and survival [88], and enhancing neuronal differentiation of NPCs [65], [89], as well as promoting neurogenesis of NSCs co-cultured with astrocytes [92].

Several previous studies have attempted to infuse NMDA or BDNF to facilitate the survival and neuronal differentiation of NPCs/NSCs [4], [63], [64], [93]. However, to date, none of these treatments have shown significant therapeutic benefits, and under certain conditions, an infusion of NMDAR agonists even results in neuronal death [94]. Among many potential reasons, an explanation for these paradoxical outcomes may be due to the rather complex actions of NMDARs and BDNF receptors. The NMDAR is a heterotetramer comprising two GluN1 subunits and two GluN2 subunits from subtypes GluN2A-GluN2D. The GluN2 subunits not only determine important biological properties, and subcellular localizations of the receptors, but also strongly influence their functional outcomes [11], [12], [95]–[97]. Native NMDARs in most neurons of the adult brain contain GluN2A and/or GluN2B subunits and these subpopulations of receptors are subcellularly segregated with GluN2A-containing and GluN2B-containing subtypes dominating synaptic and extrasynaptic compartments, respectively [90], [95]. Selective activation of synaptic (predominantly GluN2A-containing) NMDARs often results in LTP [10] which is generally associated with activation of cell survival signaling [32], [94], [98], [99] and promotes cell survival [11], [12], whereas activation of both synaptic and extrasynaptic (predominantly GluN2B-containing) or preferential activation of extrasynaptic NMDARs favours the production of LTD [10], [96] which is generally linked with the activation of cell death signaling [100]–[102] and leads to neuronal death [11], [12], [90]. Thus, it is possible that clinical infusion of NMDAR agonists, by non-specifically stimulating both synaptic and extrasynaptic NMDARs, produces cell-death associated LTD, and therefore, may not be expected to mimic LTP actions in promoting both survival and neuronal differentiation of NPCs/NSCs. Similarly, infusion of BDNF alone may also be insufficient. This could be due to the fact that BDNF may only be one of multiple neurogenesis neurotrophic factors released following LTP production, with BDNF required to act in concert with these factors to mimic LTP, thereby promoting neurogenesis.

Unlike the infusion of either NMDAR agonists or BDNF, electrical stimulation of host neurons in the brain with a LTP producing protocol can increase glutamate release from the presynaptic terminals. However, because of the presence of efficient synaptic and parasynaptic glutamate transporters, the presynaptically released glutamate is quickly uptaken before it can diffuse outside of the synapses to reach extra-synaptic (predominantly GluN2B-containing) NMDARs. Thus, a LTP producing protocol is able to preferentially activate synaptic (predominantly GluN2A-containing) NMDARs [10], [96], [97], thereby activating synaptic cell survival signaling [12], [100] and increasing neuronal release of neurotrophic factors, such as BDNF (present work). Therefore, electrical stimulation of the host brain with a LTP producing protocol may be advantageous over drug treatments in pre-conditioning the host brain environment to favor proliferation/survival and neuronal differentiation of both endogenous NPCs and exogenously transplanted NSCs.

To this end, it is important to note that such electrical stimulation-induced LTP pre-conditioning of the host environment prior to NSC transplantation is clinically feasible at present. In addition to the fact that LTP can be induced in many brain areas including the hippocampus [5], DBS has been in clinical use for many years as an effective treatment of certain brain disorders [103], and commercially available devices (stimulators) designed for DBS can easily be tuned to produce LTP-inducing parameters. In fact, a recent study has reported some beneficial effects on promoting neurogenesis of endogenous NPCs by DBS and interestingly, these authors observed that the beneficial effects were stimulation frequency dependent, being only observed at high (130 Hz), but not at low (10 Hz) stimulation frequency [104], [105]. Given that the high frequency, but not low frequency, would favor the induction of LTP [5], [106] this study may be consistent with our current observation that the induction of LTP promotes neurogenesis. In addition to the feasible use of DBS in a clinical setting, direct recording of neuronal responses to electrical stimulation has also recently become clinically obtainable [107]. Such simultaneous DBS and recordings would ensure that a LTP-producing protocol delivered by DBS induces LTP. Taken together, the evidence suggests that if the LTP-mediated neurogenesis promoting effects observed in our current study are validated with additional studies, it may be practical to use a clinically validated LTP-inducing electrical stimulation protocol to condition the brain prior to NSC transplantation. Such a protocol would be expected to significantly increase NSC survival and neuronal differentiation in the brain, and hence significantly enhance success rates and efficacy of stem cell-based cell replacement therapy.

In the present study, we have demonstrated that a simple and clinically practicable LTP-inducing electrical stimulation protocol can promote proliferation/survival and neural differentiation of both endogenous NPCs and exogenously transplanted NSCs in the brain by mechanisms involving activation of NMDARs and subsequent release of diffusible neurotrophic factors including BDNF. The current availability of techniques of DBS and recordings in clinical settings suggests that our electrical stimulation-induced LTP could be developed into a clinical relevant procedure that could be used to increase the success of stem cell therapy by either increasing the induction of endogenous progenitors or promoting the survival and neural differentiation of implanted NSCs. The success of stem cell-based cell replacement therapy for repairing the lost or injured neuronal circuitries that arise in neurodegenerative brain disorders is ultimately dependent on these cells being able to functionally integrate into the damaged host neuronal networks. By prolonging the survival period and increasing the number of newly generated neurons, the induction of LTP can be expected to facilitate their functional integration. In addition, given that the induction of LTP is highly synaptic input specific, and plays a critical role in synaptic stabilization, LTP may promote the functional integration of newly generated neurons into the host neuronal networks independent of its promotion of survival and neural differentiation. Thus, future studies along this line of investigation may facilitate not only our understanding of how the mechanisms underlying LTP promotes neurogenesis of NPCs/NSCs into functional neuronal networks in the host brain, but also its use as an clinically effective pre-conditioning procedure to increase the efficacy of stem cell-based neurological therapies.
